# Temperature Compensation Method for Piezoresistive Pressure Sensors Based on Gated Recurrent Unit

**DOI:** 10.3390/s24165394

**Published:** 2024-08-21

**Authors:** Mian Liu, Zhiwu Wang, Pingping Jiang, Guozheng Yan

**Affiliations:** 1 School of Electronic Information and Electrical Engineering, Shanghai Jiao Tong University, Shanghai 200240, China; liumian1110@gmail.com (M.L.); jpp99@sjtu.edu.cn (P.J.); 2 School of Biomedical Engineering, Shanghai Jiao Tong University, Shanghai 200240, China; gzhyan@sjtu.edu.cn

**Keywords:** temperature compensation, piezoresistive pressure sensors, gated recurrent unit, improved whale optimization algorithm, random forest

## Abstract

Piezoresistive pressure sensors have broad applications but often face accuracy challenges due to temperature-induced drift. Traditional compensation methods based on discrete data, such as polynomial interpolation, support vector machine (SVM), and artificial neural network (ANN), overlook the thermal hysteresis, resulting in lower accuracy. Considering the sequence-dependent nature of temperature drift, we propose the RF-IWOA-GRU temperature compensation model. Random forest (RF) is used to interpolate missing values in continuous data. A combination of gated recurrent unit (GRU) networks and an improved whale optimization algorithm (IWOA) is employed for temperature compensation. This model leverages the memory capability of GRU and the optimization efficiency of the IWOA to enhance the accuracy and stability of the pressure sensors. To validate the compensation method, experiments were designed under continuous variations in temperature and actual pressure. The experimental results show that the compensation capability of the proposed RF-IWOA-GRU model significantly outperforms that of traditional methods. After compensation, the standard deviation of pressure decreased from 10.18 kPa to 1.14 kPa, and the mean absolute error and root mean squared error were reduced by 75.10% and 76.15%, respectively.

## 1. Introduction

Pressure sensors are the most widely used types of sensors [1,2]. They convert the pressure of a fluid or gas into an electrical signal. According to the principle of pressure sensors, they can be divided into piezoresistive, piezoelectric, capacitive, and resonant sensors, as well as other types [3]. Due to their high sensitivity, fast dynamic response, good stability, and easy integration, piezoresistive pressure sensors have extensive applications in the automotive industry, electric machinery, environmental monitoring, smart homes, and other fields [4]. However, various application scenarios and working environments pose many challenges to pressure sensors, requiring high-precision and high-stability measurements across a wide temperature range. Unfortunately, the characteristics of piezoresistive pressure sensors are often influenced by the manufacturing process and various environmental variables, leading to nonlinearity. Among these factors, temperature significantly affects the accuracy of piezoresistive pressure sensors [5]. Temperature changes impact the piezoresistive coefficient [6], causing temperature drift. Additionally, temperature changes result in the thermal expansion and contraction of materials, leading to additional thermal stress on the pressure sensor [7]. Therefore, temperature compensation to improve the output accuracy of piezoresistive pressure sensors has become a widely discussed topic in both scientific and industrial circles.

Temperature compensation methods typically involve hardware compensation and software compensation [8]. Hardware compensation aims to enhance accuracy under different scenarios through circuit design [9], primarily utilizing built-in analog circuits to mitigate temperature drift. However, as packaging processes continue to improve, most sensors are put into use after packaging, making it challenging to make subsequent adjustments after completing the hardware compensation circuit design. External temperature changes also have an irreversible impact on components in the compensation circuit, leading to a significant increase in errors as usage time progresses [10]. Moreover, hardware compensation methods increase production costs [11] and the usage costs of sensors. As a complement and extension to hardware compensation methods, software compensation methods are increasingly utilized to enhance the overall accuracy and adaptability of piezoresistive pressure sensors.

Software compensation methods exhibit strong robustness and have lower manufacturing process requirements. These methods can be categorized into numerical computation-based and machine learning-based methods. Numerical computation-based temperature compensation methods include lookup table, surface fitting, and polynomial interpolation. Lookup table can effectively compensate for temperature drift, but the accuracy after compensation is related to the number of stored points [12]. Surface fitting is adequate for the nonlinear calibration of pressure sensor outputs [13]. Wang proposed a method based on Newton interpolation and cubic spline interpolation for online temperature compensation [14]. This method is easy to implement and has high accuracy, but solving polynomial coefficients may lead to ill-conditioned problems, reducing the reliability of the compensation model. Machine learning-based methods exhibit strong nonlinear approximation capabilities, overcoming some limitations of numerical calculation and offering new options for the high-precision temperature compensation of pressure sensors. Machine learning methods commonly employed by researchers include support vector machine (SVM), extreme learning machine (ELM), and artificial neural network (ANN). Suykens introduced the least squares support vector machine (LSSVM), transforming the inequality constraints of traditional SVM into linear equations in regularization theory [15]. A study by Li demonstrates that the backpropagation (BP) neural network temperature compensation method is effective and robust [16]. However, this method has some drawbacks, such as slow convergence, susceptibility to local optima, and uncertainty in weight thresholds. Therefore, some researchers have improved the BP neural network using different optimization algorithms, accelerating the algorithm’s speed and enhancing its applicability [8,17]. Fu developed a temperature compensation model using the radial basis function (RBF) neural network optimized by chicken swarm optimization (CSO), which exhibited sound compensation effects in piezoresistive pressure sensors [18]. Ge optimized the wavelet neural network (WNN) model and applied it for temperature compensation [19]. However, the above software compensation methods are static compensation methods that require the collection of discrete data. The required data are obtained by measuring the response voltage of the sensor under different temperatures and applied pressures. These methods neglect the coupling effect of pressure and temperature on temperature drift, significantly increasing the time for calibration.

Due to thermal hysteresis, the temperature drift of pressure sensors depends not only on the current input but also on the previous information. ANNs have failed to utilize previous information effectively, leading to lower accuracy and poor robustness. Considering temperature drift’s highly nonlinear and sequence-dependent nature, deep learning networks are particularly suitable for temperature compensation [20]. Among them, recurrent neural networks (RNNs) stand out as one of the most promising deep learning networks, performing exceptionally well in time series processing. RNNs have memory capabilities, retaining and applying previous information to current output calculations. However, traditional RNNs suffer from inherent issues of gradient vanishing and exploding gradients [21]. Some improved RNN structures, such as long–short-term memory (LSTM) and gated recurrent unit (GRU), effectively address these problems. LSTM establishes new cell states and uses gate mechanisms to filter information and manage long-term memory [22]. By introducing gate mechanisms, especially the forget gate and input gate, LSTM can better remember and utilize long-term information, thus exhibiting significant advantages in handling long sequence data and remembering long-term information [23,24]. In contrast, GRU solves the gradient problems of RNNs and uses fewer parameters, resulting in shorter training times and making them suitable for processing time series data [25]. In recent years, RNNs have begun to be applied in temperature compensation for various sensors. However, there is limited research on applying RNNs to compensate for temperature compensation in pressure sensors.

To address this research gap, we propose a temperature compensation method based on the RF-IWOA-GRU model. This model first preprocesses the actual pressure data using random forest (RF), filling in the missing values in the continuous data. In response to the challenge of traditional neural networks struggling to utilize previous information effectively, we incorporate temperature, the rate of temperature change, and the output of the pressure sensor as input variables for GRU. Furthermore, we optimize the hyper-parameters of GRU using the improved whale optimization algorithm (IWOA), significantly improving the model’s training efficiency and prediction performance. Ultimately, experimental validation demonstrates the effectiveness of the RF-IWOA-GRU model. This method enhances the measurement accuracy of pressure sensors, shortens the time required for calibration, and provides a reference for the temperature compensation of other types of sensors.

## 2. Failure Mechanism of Pressure Sensors

### 2.1. Introduction of Piezoresistive Pressure Sensors

**Figure 1:**
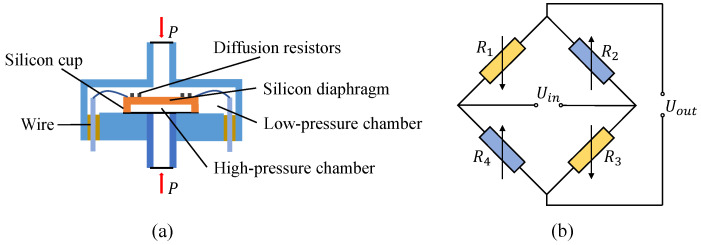
Schematic diagram of the pressure sensor. (**a**) Structure of piezoresistive pressure sensor. (**b**) Wheatstone bridge.

The structure of the piezoresistive pressure sensor [26,27] used in this study is shown in Figure 1a. The principle of the sensor is based on the piezoresistive effect of monocrystalline silicon. The core component of the sensor is a silicon diaphragm placed on a circular silicon cup. The diaphragm separates two chambers: the high-pressure chamber exposed to the measured pressure and the low-pressure chamber exposed to the standard atmospheric pressure as the reference pressure. Four diffused resistors are attached to the diaphragm, one pair experiencing compressive stress and the other experiencing tensile stress. When the pressure difference between the two chambers causes deformation of the diaphragm, the resistance changes accordingly, leading to an imbalance in the bridge and conversion of the pressure signal into a proportional electrical signal. By measuring the voltage output of the bridge, the pressure difference acting on the diaphragm can be determined, and thus, the measured pressure value can be calculated. Figure 1b shows the equivalent circuit diagram of the piezoresistive pressure sensor [28,29].

According to the principle of the Wheatstone bridge [30], the output voltage of the piezoresistive pressure sensor is given by the following:

(1) $$U_{\mathrm{out}\;}=\frac{\left(R_{2}\times R_{4}-R_{1}\times R_{3}\right)}{\left(R_{1}+R_{2}\right)\left(R_{3}+R_{4}\right)}\times U_{\mathrm{in}\;}$$

where $U_{\mathrm{in}}$ is the constant excitation power supply, and $U_{\mathrm{out}}$ is the output voltage.

When the pressures in the two chambers are equal, the relationship between the resistances in the arms of the Wheatstone bridge is $R_{1}\times R_{3}=R_{2}\times R_{4}$, resulting in the bridge being balanced and the output voltage, $U_{\mathrm{out}\;}$, being zero. However, when there is a pressure difference across the silicon diaphragm, the diaphragm deforms, causing the four resistances to change, and the bridge becomes unbalanced, ultimately generating an output voltage:

(2) $$U_{\mathrm{out}}=\frac{\left(R_{2}+\Delta R_{2}\right)\left(R_{4}+\Delta R_{4}\right)-\left(R_{1}-\Delta R_{1}\right)\left(R_{3}-\Delta R_{3}\right)}{\left[\left(R_{1}-\Delta R_{1}\right)+\left(R_{2}+\Delta R_{2}\right)\right]\left[\left(R_{3}-\Delta R_{3}\right)+\left(R_{4}+\Delta R_{4}\right)\right]}\times U_{\mathrm{in}\;}$$

According to the piezoresistive effect, if process errors are disregarded, and it is assumed that $R_{1}=R_{2}=R_{3}=R_{4}=R$, with corresponding resistance changes being identical and the stress experienced by the resistors being the same, then the resistance change induced by biaxial stress is given by the following:

(3) $$\frac{\Delta R}{R}=\pi_{l}\sigma_{l}+\pi_{t}\sigma_{t}$$

where $\pi_{l}$ and $\pi_{t}$ are the longitudinal and transverse piezoresistance coefficients, and $\sigma_{l}$ and $\sigma_{t}$ are the longitudinal and transverse stresses, respectively.

In summary, Equation (2) can be simplified into the following:

(4) $$U_{\mathrm{out}\;}=I_{\mathrm{in}\;}\times R\times \left(\pi_{l}\sigma_{l}+\pi_{t}\sigma_{t}\right)$$

### 2.2. Temperature Effects of Pressure Sensors

Due to unpredictable errors in the manufacturing process of pressure sensors, it cannot be guaranteed that the resistance of each resistor in the Wheatstone bridge is equal. The resistance and silicon piezoresistive coefficient with temperature change are given by the following [31]:

(5) $$R=R_{0}\left(1+\alpha \Delta T\right)$$

where $R_{0}$ is the reference resistance, α is the temperature coefficient of resistance, and ΔT is the temperature change.

Furthermore, the piezoresistive coefficient π is influenced by the silicon doping level and temperature, with temperature changes leading to variations in the piezoresistive coefficient [32]. Additionally, heat generates thermal expansion and contraction when the resistor is powered. The packaging material of the pressure sensor also changes due to temperature, resulting in additional thermal stress Δσ.

In summary, changes in actual pressure and temperature will both cause nonlinear variations in the output voltage, $U_{\mathrm{out}}$. The coupling effect between pressure and temperature makes these variations even more unpredictable. Moreover, other factors, such as the hysteresis of the electronic components and the different impurity doping concentrations, can lead to more complex variations in the temperature drift.

### 2.3. Factors for Temperature Compensation

In environments with changing temperatures, the error of piezoresistive pressure sensors is mainly caused by thermal zero drift, changes in thermal sensitivity, and thermal hysteresis [33]. For temperature compensation in pressure sensor research, the environment can be comprehensively described by the temperature itself, the rate of temperature change, and the temperature gradient [34,35].

The pressure sensor and the probe of the thermometer are placed inside a sealed metal container in our experiment. However, due to the thermal time constants of the pressure sensor and probe varying inconsistently with temperature changes, this results in uneven heat conduction within the metal container. This inconsistency creates complex thermal hysteresis between the actual temperature of the pressure sensor and the collected temperature. The effects disrupt the coupling relationship between temperature and pressure, introducing errors in pressure measurement.

Considering the above factors, the input variables of the temperature compensation model of the pressure sensor include temperature, *T*; the rate of temperature change, $\dot{T}$; and the output of the pressure sensor, $U_{\mathrm{out}}$. The temperature gradient, ∇T, causes an asymmetric change in the temperature field, and the effect mechanism is similar to the rate of temperature change. However, its measurement requires multiple thermometers. We only use one thermometer in the experiment, so it is not considered for now. In addition, the second-order rate of temperature change, $\ddot{T}$, is not included as an input variable because *T* and $\dot{T}$ are sufficient for capturing the temperature variation trends, while also reducing the model’s complexity. Furthermore, the low temperature sampling rate may lead to an inaccurate computation of $\ddot{T}$, potentially introducing additional noise and instability.

Through analysis, we know that the purpose of temperature compensation is to establish the following relationship:

(6) $$\hat{P}=f\left(U_{\mathrm{out}\;},T,\dot{T}\right)$$

where $\hat{P}$ is the predicted pressure value. f(·) is an unknown complex function that relates to the characteristics of the pressure sensor and external environmental factors, which we will model using gated recurrent unit networks in Section 3.

## 3. Compensation Algorithms and Model

Based on the above discussion, it can be concluded that the temperature drift of the pressure sensor depends on the current input and previous information. Traditional temperature compensation methods do not utilize previous information, leading to poor predictive performance and weak robustness. Gated recurrent unit (GRU) networks can remember historical information by changing their internal state, which allows the information to be backpropagated smoothly. For modeling with GRU, a continuous time series dataset is indispensable for improving prediction accuracy. However, the actual pressure data collected in experiments contain missing values. Therefore, we utilize random forest (RF) to predict and fill these missing values using other relevant features. Considering the model’s performance is significantly influenced by the hyper-parameters of GRU, a meta-heuristic algorithm is adopted to optimize the hyper-parameters. Compared with other traditional meta-heuristic algorithms, the improved whale optimization algorithm (IWOA) boasts the advantages of stable convergence, minimal parameters, and straightforward implementation.

### 3.1. Random Forest

Random forest (RF) is a versatile machine learning method that extends the classification and regression tree (CART) approach. It is a supervised learning algorithm that constructs an ensemble of decision trees known as a “forest”, typically trained using the bagging process. RF applies to both regression and classification problems [36]. In regression problems, RF combines multiple simple trees, which vote for their responses and average them to estimate the dependent variable [37]. Data and variables can be iteratively sampled randomly using bootstrap sampling to generate a forest of regression trees. The predictions of RF are the average predictions of these trees:

(7) $$\hat{y}=\frac{1}{K}\sum_{k=1}^{K}{\hat{y}}_{k}$$

where *k* indexes the individual trees in the forest.

RF uses out-of-bag errors to calculate the relative importance of feature variables, thereby enhancing regression accuracy. Implementation of RF depends on the regularization of decision trees and stopping parameters. Parameters of the decision tree model include the maximum number of trees that must be grown in the forest and the number of variables randomly selected in each node.

### 3.2. Gated Recurrent Unit Networks

While traditional time series prediction methods are typically designed for steady-state signals, the reality is that most signals are non-steady-state. Consequently, utilizing neural networks to address time series problems has emerged as a significant approach [38]. Unlike traditional neural networks, which can only handle a single input signal without considering the mutual influence between the input at the previous moment and the output at the next moment, time series problems require us to account for the impact of past inputs on future outputs. The GRU has gained widespread acceptance as an approach capable of capturing temporal dependencies due to its high computational efficiency and accurate time feature extraction [39].

**Figure 2:**
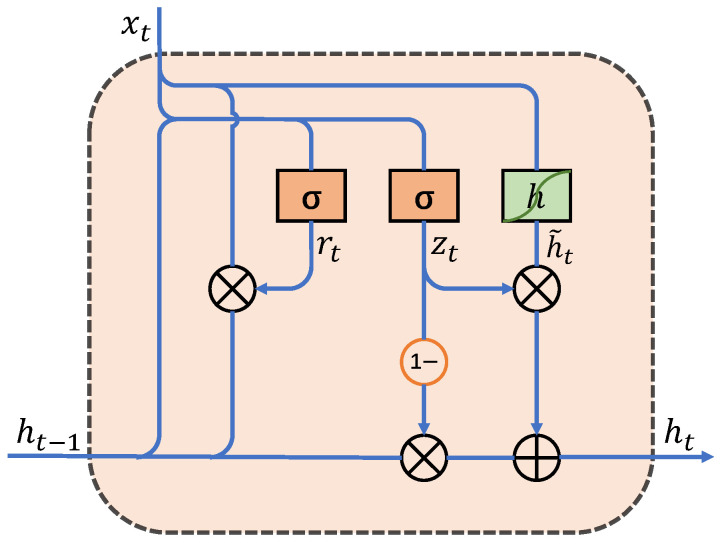
The schematic diagram of GRU.

The GRU model comprises the update gate, $z_{t}$, and the reset gate, $r_{t}$. The update gate controls the information carried from the previous state to the current state, while the reset gate determines the integration of new input information with the previous memory [40]. Figure 2 illustrates the schematic diagram of the GRU. The two gating states are derived from a hidden state, $h_{t-1}$, transmitted from the previous node and a current input node, $x_{t}$, and can be expressed as follows:

(8) $$r_{t}=\sigma \left(W_{xr}x_{t}+W_{hr}h_{t-1}+b_{r}\right)$$

(9) $$z_{t}=\sigma \left(W_{xz}x_{t}+W_{hz}h_{t-1}+b_{z}\right)$$

Furthermore, the reset and update gates together contribute to the derivation of the final hidden state:

(10) $$\begin{array}{cc} \; & {\tilde{h}}_{t}=\tanh\left(W_{hx}x_{t}+W_{hh}\left(r_{t}⊙h_{t-1}\right)+b_{h}\right) \end{array}$$

(11) $$\begin{array}{cc} \; & h_{t}=\left(1-z_{t}\right)⊙h_{t-1}+z_{t}⊙\tilde{h_{t}} \end{array}$$

where *W* is the weight matrix connecting different structures; *b* is the offset vector of the corresponding structures; *x*, *h*, *r*, and *z* are the input, hidden state, reset gate, and update gate, respectively.

The sequences of temperature, the rate of temperature change, and the output of the pressure sensor are taken as the input variables of GRU, while the actual pressure is taken as the output variable. GRU contains three hyper-parameters that affect the accuracy of the model, including the initial learning rate, the batch size, and the number of neurons. Here, the IWOA is used to find the optimal parameters of the GRU.

### 3.3. WOA and IWOA

#### 3.3.1. Whale Optimization Algorithm

WOA is a meta-heuristic algorithm proposed by Mirjalili, inspired by the behavior of humpback whales in capturing prey in the wild [41]. In this algorithm, the position of each whale represents the problem solution, whereas the position of the prey refers to the optimal solution of the problem. During hunting, whales randomly choose one of the following two parts with a 50% probability.

- Contraction Encircling
  This process imitates the behavior of whales surrounding their prey through circular movement. The way the whales shrink their enclosures is modulated by $\left|A\right|$.
  (12) $$A=a\left(2r-1\right)$$
  where *r* is the random number in the interval $\left[0,1\right]$, and *a* is the adjustment factor.
  At $\left|A\right|<1$, the algorithm performs a local search.
  (13) $$\begin{array}{cc} \; & a=-2\left(\frac{t}{T}-1\right) \end{array}$$
  (14) $$\begin{array}{cc} \; & C=2r \end{array}$$
  (15) $$\begin{array}{cc} \; & D^{\prime }=\left|C\cdot X_{\left(t\right)}^{*}-X_{\left(t\right)}\right| \end{array}$$
  (16) $$\begin{array}{cc} \; & X_{\left(t+1\right)}=X_{\left(t\right)}^{*}-A\cdot D^{\prime } \end{array}$$
  where $D^{\prime }$ is the distance between the current whale position and the best whale position, $X_{\left(t\right)}^{*}$ and $X_{\left(t\right)}$ are the optimal position and the current individual position at the *t* iteration, respectively, *C* is the perturbation coefficient, and *T* is the maximum number of iterations.
  At $\left|A\right|\geq 1$, the algorithm performs a global search.
  (17) $$\begin{array}{c} D_{R}=\left|C\cdot X_{R(t)}-X_{\left(t\right)}\right| \end{array}$$
  (18) $$\begin{array}{c} X_{\left(t+1\right)}=X_{R(t)}-A\cdot D_{R} \end{array}$$
  where $X_{R(t)}$ is the random whale position in the current iteration, and $D_{R}$ is the distance between the random whale position and the current whale position.
- Spiral Updating position
  This process imitates the behavior of whales encircling a target through spiral movement.
  (19) $$\begin{array}{cc} D^{*} & =\left|X_{\left(t\right)}^{*}-X_{\left(t\right)}\right| \end{array}$$
  (20) $$\begin{array}{cc} X_{\left(t+1\right)} & =D^{*}\cdot e^{bl}\cdot \cos\left(2\pi l\right)+X_{\;\;(t)}^{*} \end{array}$$
  where $D^{*}$ is the distance between the current and best whale positions, *b* is the spiral shape coefficient, and *l* is the random number in the interval [−1,1].

In summary, the hunting behavior of whales can be summarized by the following mathematical model:

(21) $$X_{\left(t+1\right)}=\left\{\begin{array}{c} \left\{\begin{array}{c} X_{\left(t\right)}^{*}-A\cdot D^{*},\;\;\left|A\right|<1\\ X_{R(t)}-A\cdot D_{R},\left|A\right|\geq 1 \end{array},p<0.5\right.\\ \;\;D^{*}\cdot e^{bl}\cdot \cos\left(2\pi l\right)+X_{\left(t\right)}^{*}\;,p\geq 0.5 \end{array}\right.$$

where *p* is the random number in [0,1]. When p<0.5, the contraction surrounding position is selected, and when p≥0.5, the spiral update position is selected.

#### 3.3.2. Improved Whale Optimization Algorithm

Although the traditional WOA has advantages such as high efficiency, few control parameters, and the ability to avoid local optima, it has certain limitations regarding convergence speed and precision. To address this issue, we propose an improved version of WOA, which mainly focuses on the following two aspects.

- Chebyshev Chaotic Map
  The Chebyshev chaotic map generates chaotic sequences through the repeated application of iterative functions. It exhibits nonlinearity, high sensitivity to initial values, excellent ergodicity, and unpredictability [42]. This map is frequently used for population initialization to enhance algorithmic diversity and randomness [43]. Based on the comparison of chaotic optimization algorithms, the Chebyshev chaotic map has a large Lyapunov exponent, which means its chaotic nature is pronounced, and its degree of chaos is high. Furthermore, considering the optimal, mean, and standard values, the Chebyshev chaotic map exhibits stability and high applicability. Therefore, considering all these factors, we chose the Chebyshev chaotic map to optimize the traditional whale algorithm. The formula is as follows:
  (22) $$x_{n+1}=\cos\left(\lambda \cdot \cos^{-1}x_{n}\right)$$
  where $x_{n}\in \left(-1,1\right)$ and λ are control parameters.
- Adaptive Weight
  In the case of contracting the encircling and spiral updating positions, a large weight value should be selected at the initial stage of the algorithm to improve the optimization speed and global search ability of the algorithm [44]. As the algorithm iterates continuously, the weight values should gradually decrease to reduce the fluctuation amplitude of the search and improve the convergence accuracy of the algorithm. Given the above situation, adaptive weight ξ is introduced to optimize the algorithm so that the contraction encircling and spiral updating positions are adjusted according to the current iteration degree. The mathematical expression of ξ can be expressed as follows:
  (23) $$\xi =B_{1}+B_{2}\cdot \sin\left(t\cdot \frac{\pi }{5}\right)\cdot e^{\left(-\frac{t}{T}\right)}$$
  where $B_{1}$ and $B_{2}$ are constants. ξ decreases with the number of iterations of the whale population. With the following, we can update Equation (21) to become the following:
  (24) $$X_{\left(t+1\right)}=\left\{\begin{array}{c} \left\{\begin{array}{c} X_{\left(t\right)}^{*}-\xi \cdot A\cdot D^{*},\;\;\left|A\right|<1\\ \xi \cdot (X_{R(t)}-A\cdot D_{R}),|A|\geq 1 \end{array},p<0.5\right.\\ \xi^{2}\cdot D^{*}\cdot e^{bl}\cdot \cos\left(2\pi l\right)+\xi \cdot X_{\left(t\right)}^{*},p\geq 0.5 \end{array}\right.$$

### 3.4. RF-IWOA-GRU Model

As discussed above, the design of hyper-parameters for GRU is essential. The IWOA possesses global solid search capability, with a fast convergence speed that allows it to quickly find the global optimal solution and avoid falling into the local optimal solution. In this study, we utilize the IWOA to search for hyper-parameters such as the initial learning rate, batch size, and number of neurons in GRU until the desired prediction accuracy is achieved.

**Figure 3:**
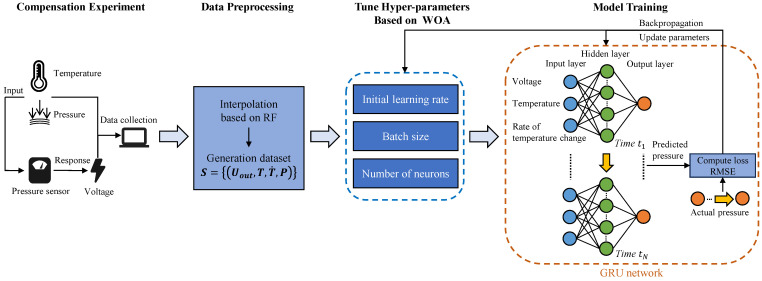
Architecture of RF-IWOA-GRU network model.

As time series data, the outputs of the pressure sensor are characterized by instability, periodic uncertainty, and nonlinearity, and are influenced by various factors. We use the advantages of GRU in time series analysis to establish a temperature compensation model. The RF-IWOA-GRU model is constructed by combining RF, the IWOA, and GRU to align the model’s structure with the output characteristics of the pressure sensor. Several hyper-parameters controlling the structure and model parameters in GRU are optimized through the IWOA. Figure 3 illustrates the structure of the RF-IWOA-GRU model.

The steps for modeling with the RF-IWOA-GRU are as follows.

(1) Experiment. Vary and record the ambient temperature and actual pressure while continuously collecting the corresponding output voltage from the pressure sensor.

(2) Preprocess data. Use the random forest to predict and fill in missing values. Combine the output of the pressure sensor with the temperature and the rate of temperature change as inputs to the model.

(3) Initialize and adjust multiple sets of hyper-parameters. The original sets are generated randomly through the Chebyshev chaotic map, including the initial learning rate, the batch size, and the number of neurons. Each set of hyper-parameters represents a different structure of GRU. The loss function is used as the fitness function of the IWOA. The IWOA adjusts hyper-parameters through this function until the conditions are met.

(4) Train the model. Train the GRU networks using the dataset. Then, the trained model will be utilized for prediction, compensating for the error of the pressure sensors.

## 4. Experiment and Results Analysis

### 4.1. Experiment and Data Collection

**Figure 4:**
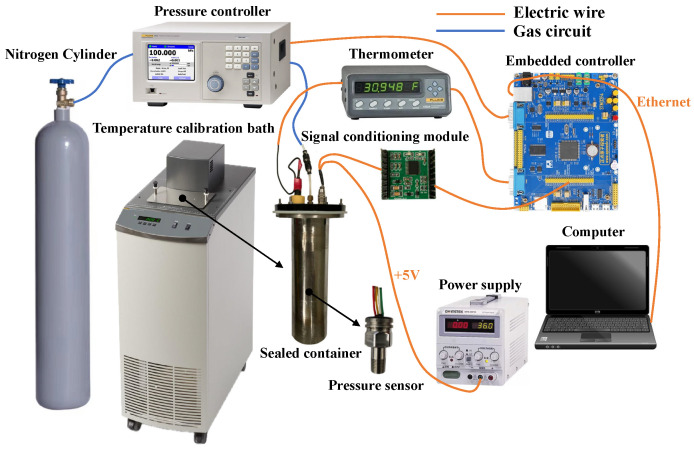
Experimental environment and equipment.

Temperature experiments were carried out to demonstrate the necessity of the IWOA-GRU temperature compensation model based on RF and to examine the temperature characteristics of the pressure sensor. The experimental environment and equipment are shown in Figure 4. In this experiment, the pressure sensor and thermometer probe were securely placed inside a sealed metal container, which served as the controlled environment for our measurements. This metal container was placed in a temperature calibration bath to regulate the surrounding environmental temperature. Pressure within the container was controlled via a pressure controller connected to a nitrogen cylinder, allowing us to create various pressure conditions for the sensor. The primary goal of the experiment was to evaluate the effectiveness of the compensation method. In practical applications, it is recommended to use a higher-integrated temperature sensor positioned at an appropriate distance from the pressure sensor. During the experiment, temperature and actual pressure data were collected using an embedded controller. When the piezoresistive pressure sensor was powered, it produced an output voltage that was sent to a signal-conditioning module. This module amplified the voltage and converted it into digital data at a frequency of 150 Hz using a 24-bit ADC. The digital data were then stored and made available to the embedded controller, which transmitted them to a computer via Ethernet for data processing and neural network training. In practical scenarios, once the computer receives the output voltage and corresponding temperature data, the pre-trained model processes this information to predict the pressure value in real time.

**Figure 5:**
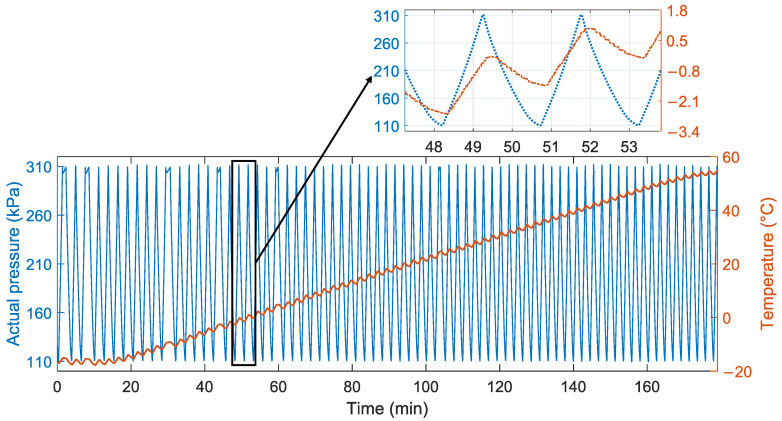
The time series data.

To improve calibration accuracy and reduce calibration time, we conducted an experiment capable of collecting time series data, the experimental process of which is illustrated in Figure 5. We first kept the temperature calibration bath at −20 °C for 20 min to ensure the temperature was stable and then heated it at a rate of 0.5 °C/min to 60 °C. Simultaneously, the pressure controller continuously cycled the actual pressure between 110 kPa and 310 kPa until the experiment was concluded. Notably, during the pressure cycling process, airflow inside the container caused temperature changes of about ±2 °C. These fluctuations mimicked real-world thermal noise, introducing random temperature drift that helped us evaluate the robustness of the model. To avoid chance factors, we conducted four experiments and found that the drift trends were consistent. From these experiments, we selected a dataset ranging from 60 °C to −20 °C for testing the model.

**Figure 6:**
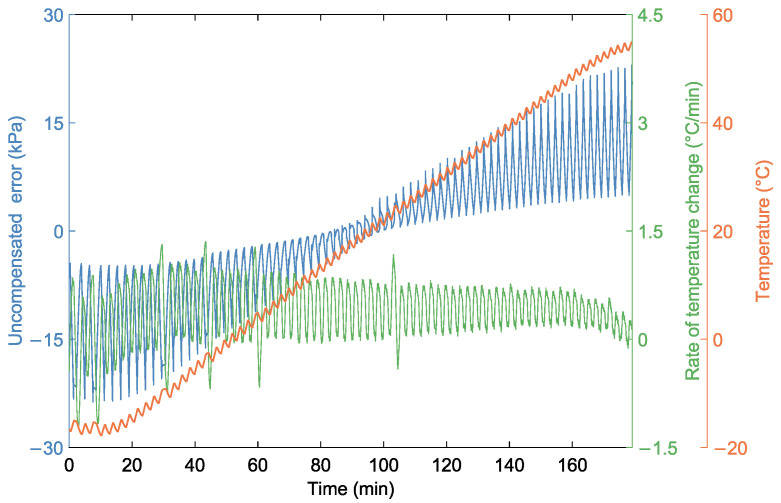
The relationship between uncompensated error and temperature.

**Figure 7:**
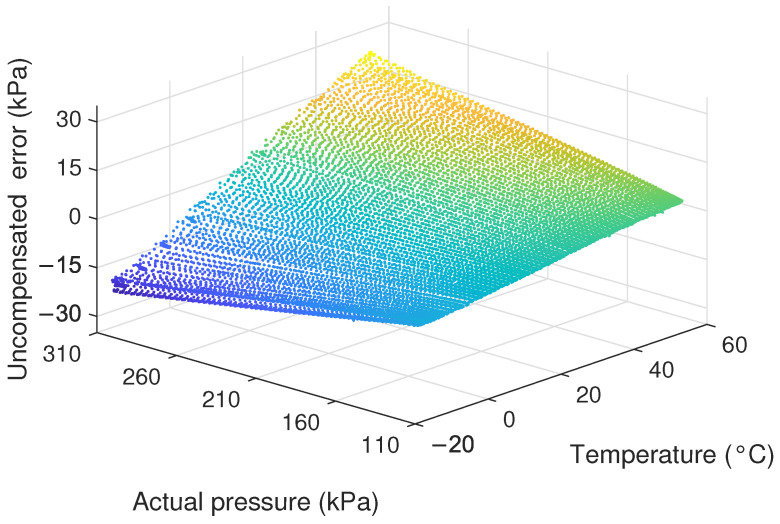
The errors at different temperatures and actual pressures.

Additionally, we selected sensor output voltages of 160 kPa and 260 kPa, measured at 20 °C, as reference points to establish a linear conversion relationship between output voltage and pressure. The sensor output voltages, recorded under various conditions, were linearly converted to corresponding pressure values. The uncompensated error was determined by subtracting the converted pressure values from the actual pressure values. Figure 6 shows the relationship between uncompensated error and temperature. We can see that the error follows the same trend as the temperature and is affected by the rate of change in temperature. In addition, Figure 7 shows the errors at different temperatures and actual pressures. It is evident that due to the presence of thermal sensitivity, which varies with temperature, the error increases continuously with the rise in actual pressure.Therefore, it is necessary to establish a temperature compensation model.

### 4.2. Data Preprocessing

#### 4.2.1. Calculation of the Rate of Temperature Change

An effective rate of temperature change must be calculated to serve as input for the compensation model. The rate of temperature change is represented as the derivative of temperature with respect to time, denoted as $\frac{\partial T}{\partial t}$. Since the temperature changes at a slow rate of 0.5 °C/min (0.0083 °C/s), the rate of temperature change calculated using only adjacent points is close to zero. Therefore, we propose a rate of temperature change calculation method based on the moving average (MA). The MA involves applying a sliding window to a data sequence and calculating the average of the data points within the window to smooth the data. The moving average temperature, $\bar{T}\left(i\right)$, at time *i* is calculated as follows:

(25) $$\bar{T}\left(i\right)=\frac{1}{k}\sum_{j=i-\frac{k}{2}}^{i+\frac{k}{2}}T\left(j\right)$$

where *k* is the window length. To reduce noise and fluctuations in temperature measurements, we set the window length *k* to 120.

Therefore, the rate of temperature change, $\dot{T}\left(i\right)$, can be given by the following:

(26) $$\dot{T}\left(i\right)=\frac{\bar{T}\left(i+1\right)-\bar{T}\left(i-1\right)}{2\cdot \Delta t}$$

where Δt is the sampling interval, and in our experiment, Δt=1 s.

#### 4.2.2. Interpolation of Missing Actual Pressure Data Values

In our experiment, the pressure controller required a certain amount of time to switch between increasing and decreasing pressure, during which serial communication to transmit actual pressure data was not possible. As a result, whenever the actual pressure reached 110 kPa or 310 kPa, the actual pressure sequence experienced three consecutive missing values. These missing values disrupted the temporal continuity of the data, making it challenging for the GRU networks to capture long-term dependencies in the time series. Additionally, missing values hindered gradient propagation, making it difficult for the model to converge and generalize, ultimately reducing the predictive accuracy of the model.

RF effectively handles non-linear relationships, exhibits robustness against outliers and noise, and utilizes all available features. Unlike traditional methods such as linear or spline interpolation, RF provides more accurate imputation by accounting for the correlations between multiple variables, resulting in more reliable estimates of missing values. Therefore, RF is utilized to interpolate the missing values before training the GRU networks. We first partitioned the data, using temperature and the output of the pressure sensor as inputs to the model, and trained the model with data that did not contain missing values. Then, we used the trained model to predict and fill in the missing values in the actual pressure data. Although the true values corresponding to the missing values could not be obtained, the interpolated data were observed to follow the original trend. Moreover, the variance before and after interpolation was calculated and found to show no significant change, indicating the consistency of the data, thereby validating the effectiveness of RF.

### 4.3. Modeling and Training

After preprocessing the input and output data, a model was constructed using temperature, the rate of temperature change, and the output of the pressure sensor as input features to predict corresponding actual pressure values through a regression output layer. A GRU, dropout, and fully connected layers between the input and output layers were optimized using the Adam optimizer. Considering computational efficiency and training time, the GRU was trained for 60 epochs.

Model training was conducted alongside the IWOA search for optimal hyper-parameters. In the IWOA, we set the number of whales to 15 and the maximum number of iterations to 50. The initial ranges of the parameters optimized in IWOA and the final search results are shown in Table 1. This completed the training of the RF-IWOA-GRU model, and the optimal performance neural network was saved.

### 4.4. Results and Discussions

**Figure 8:**
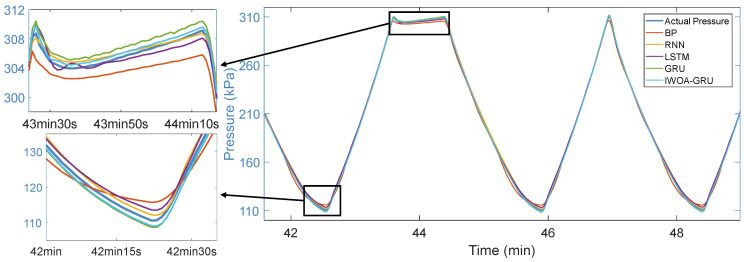
The actual pressure and the predictions of each model.

Next, to validate the effectiveness and accuracy of the proposed RF-IWOA-GRU model, comparisons were made with other models, including GRU, LSTM, RNN, and BP neural network models. Each network model’s parameters were appropriately allocated. Across the entire training set, all models were generally able to predict the actual pressure trend. To more clearly compare performance, Figure 8 shows the actual pressure and the predictions of each model over a segment of the training set. Overall, the RF-IWOA-GRU model outperforms all other models in prediction accuracy.

To quantitatively compare the prediction performance of the RF-IWOA-GRU model with that of other neural network models, mean absolute error (MAE), mean absolute percentage error (MAPE), and root mean square error (RMSE) were used as error metrics. The smaller these error metrics, the better the model’s compensation effect. The definitions of these error metrics are as follows:

(27) $$\;\mathrm{MAE}\;=\frac{1}{n}\sum_{i=1}^{n}\left|{\hat{y}}_{i}-y_{i}\right|$$

(28) $$\;\mathrm{MAPE}\;=\frac{1}{n}\sum_{i=1}^{n}\left|\frac{{\hat{y}}_{i}-y_{i}}{{\hat{y}}_{i}}\right|\times 100\%$$

(29) $$\;\mathrm{RMSE}\;=\sqrt{\frac{1}{n}\sum_{i=1}^{n}{\left({\hat{y}}_{i}-y_{i}\right)}^{2}}$$

where ${\hat{y}}_{i}$ is the predicted value, $y_{i}$ is the actual value, and *n* is the number of samples.

From Table 2, it can be seen that the RF-IWOA-GRU model outperforms the other models. Specifically, the proposed RF-IWOA-GRU model reduces the MAE by 68.36%, 62.99%, 32.44%, and 28.77%, and the RMSE by 68.43%, 61.92%, 32.85%, and 24.15% compared to other models. This indicates that the RF-IWOA-GRU model can better extract the characteristics of the pressure sensor and has more minor prediction deviations.

Additionally, the prediction performance of the GRU and LSTM models is relatively close. Compared to the LSTM model, the GRU model omits the separate output gate, using only the update and reset gates to solve the gradient vanishing and exploding problems, reducing computational cost requirements and improving computational efficiency. Among the five models, the BP neural network performs the worst, indicating its lack of capability in handling time series data and capturing the intrinsic relationship between temperature drift data caused by thermal hysteresis. Moreover, the performance of RNN is significantly lower compared to that of the GRU and LSTM models, as RNN fails to capture the long-term dependency of temperature drift on previous information. This result also highlights the importance of long-term memory in modeling. Notably, the IWOA-GRU model’s prediction performance surpasses that of GRU, demonstrating that the IWOA can find better hyper-parameters, and the GRU model is sensitive to hyper-parameter settings. The IWOA can prevent GRU from converging to local optima and enhance the model’s robustness and generalization ability.

**Figure 9:**
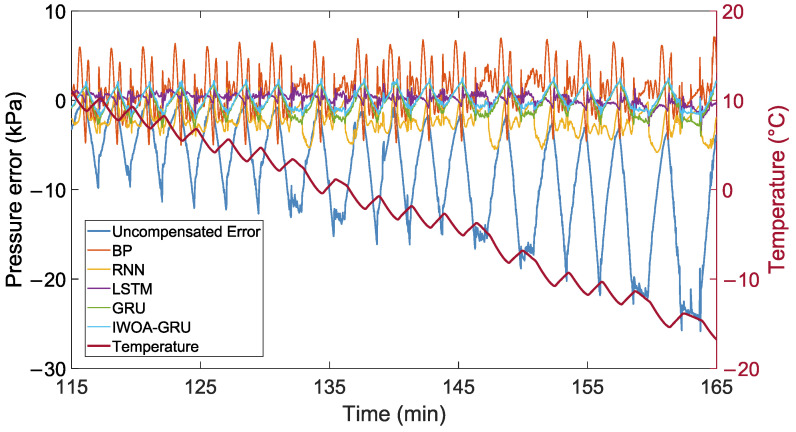
Uncompensated and compensated errors on test set.

The same experiments and quantitative analyses were conducted using the test set to validate the proposed model’s effectiveness further. Figure 9 shows the uncompensated error and the error after compensation of each model on the test set. The error metrics for each model are shown in Table 3. The table shows that the RF-IWOA-GRU model performs the best, with model performance rankings similar to those in the training set results. Notably, compared to the uncompensated error, the MAE after temperature compensation using the proposed model decreased by 75.10%, and the RMSE decreased by 76.15%. Additionally, the standard deviation of the compensated pressure decreased from 10.18 kPa to 1.14 kPa.

In summary, the proposed RF-IWOA-GRU model was validated for the temperature compensation of the pressure sensors. The comparison with other models indicated that the RF-IWOA-GRU model excels in prediction accuracy and computational efficiency.

## 5. Conclusions

In this study, we proposed a novel temperature compensation method for piezoresistive pressure sensors based on GRU optimized by the IWOA. Our experiments demonstrated that the RF-IWOA-GRU model outperforms traditional compensation models regarding prediction accuracy and computational efficiency.

The GRU model, with its simpler structure compared to that of LSTM and its ability to handle long-term dependencies, proved effective in capturing the intrinsic relationship between the output of the sensor and temperature. The integration of the IWOA with GRU further enhanced the model’s performance by optimizing its hyper-parameters, thereby preventing the model from converging to local optima and improving its robustness and generalization ability. In conclusion, the proposed RF-IWOA-GRU model provides a robust and efficient solution for temperature compensation in piezoresistive pressure sensors and is a reference for other types of sensors.

## Figures and Tables

**Table 1:** Model setting parameters.

Model Parameter	Numerical Value
Initial learning rate	0.001–0.01
Batch size	10–250
Number of neurons	2–50

**Table 2:** Prediction error on the training set.

Model	MAE	MAPE	RMSE
BP	2.089	0.0129	2.616
RNN	1.786	0.0103	2.169
LSTM	0.976	0.0063	1.230
GRU	0.928	0.0060	1.089
IWOA-GRU	0.661	0.0043	0.826

**Table 3:** Prediction error on the test set.

Model	MAE	MAPE	RMSE
Uncompensated	8.260	0.0402	10.18
BP	4.259	0.0267	5.403
RNN	3.561	0.0198	4.244
LSTM	2.896	0.0156	3.616
GRU	2.787	0.0172	3.338
IWOA-GRU	2.027	0.0124	2.428

## Data Availability

The data presented in this study are available on request from the corresponding author. The data are not publicly available due to privacy restrictions.

## Abbreviations

The following abbreviations are used in this manuscript:

SVM	Support vector machine
ANN	Artificial neural network
RF	Random forest
GRU	Gated recurrent unit
WOA	Whale optimization algorithm
IWOA	Improved whale optimization algorithm
ELM	Extreme learning machines
LSSVM	Least squares support vector machine
BP	Backpropagation
RBF	Radial basis function
CSO	Chicken swarm optimization
WNN	Wavelet neural network
RNN	Recurrent neural network
LSTM	Long–short-term memory
CART	Classification and regression tree
MA	Moving average
MAE	Mean absolute error
MAPE	Mean absolute percentage error
RMSE	Root mean square error
